# Hepatitis B Virus-Related Glomerulonephritis: Not a Predominant Cause of Proteinuria in Korean Patients with Chronic Hepatitis B

**DOI:** 10.1155/2015/126532

**Published:** 2015-02-18

**Authors:** Jeong-Ju Yoo, Jeong-Hoon Lee, Jung-Hwan Yoon, Minjong Lee, Dong Hyeon Lee, Yuri Cho, Eun Sun Jang, Eun Ju Cho, Su Jong Yu, Yoon Jun Kim, Hyo-Suk Lee

**Affiliations:** ^1^Department of Internal Medicine and Liver Research Institute, Seoul National University College of Medicine, Seoul National University Hospital, Seoul 110744, Republic of Korea; ^2^Department of Internal Medicine, Seoul National University College of Medicine, Seoul National University Bundang Hospital, Seongnam City, Gyeonggi-do, Seoul 110744, Republic of Korea

## Abstract

*Background/Aims*. Hepatitis B virus (HBV) can form immune complexes which may result in various types of glomerulonephritis (GN). However, proteinuria can occur because of other kidney diseases besides HBV-related GN (HBV-GN). The aim of this study is to elucidate the causes of proteinuria and report on the clinical outcomes of HBV-GN. *Methods*. We reviewed the medical records of patients positive for serum hepatitis B surface antigen who underwent renal biopsies due to proteinuria at a tertiary medical center in Korea. *Results*. A total of 55 patients were included. HBV-GN was diagnosed in 20 (36.4%) of the patients by confirming the presence of immune complexes (12 of 13 membranoproliferative glomerulonephritis, 7 of 8 membranous glomerulonephritis, and 1 of 13 immunoglobulin A nephropathy). Twenty-one patients had other types of GN. A total of 13 (65%) HBV-GN patients were treated with antiviral agents for a median of 11 months. However, the degrees of proteinuria were not significantly reduced in the antiviral intervention group when compared to the control group. *Conclusions*. Proteinuria can be caused by various glomerular diseases and HBV-GN accounts for one-third of total GN cases. Well-designed prospective study is needed to assess whether antiviral therapy against HBV infection may improve the prognosis of HBV-GN.

## 1. Introduction

Marked differences are seen in the epidemiology of hepatitis B virus (HBV) infection between various regions [1]. In Korea, the prevalence of HBV infection is as high as approximately 5%, and HBV infection is the most important cause of chronic liver disease including liver cirrhosis and hepatocellular carcinoma [2]. HBV infection can also manifest extrahepatically with diseases such as polyarteritis nodosa, a systemic necrotizing vasculitis mediated by immune complexes [3]. HBV-related glomerulonephritis (HBV-GN) is one of the extrahepatic manifestations of chronic hepatitis B (CHB) infection and is an uncommon, but problematic, complication of chronic hepatitis B (CHB). HBV has been reported to form immune complexes which can deposit in the glomeruli of the kidney and result in various types of glomerulonephritis (GN). HBV-GN may present with mild to moderate proteinuria, nephrotic syndrome, and hematuria [4].

Diagnosis of HBV-related GN (HBV-GN) is made through a series of serological markers of HBV, as well as by renal biopsy. The most common findings in renal histology are membranous glomerulonephritis (MGN), membranoproliferative glomerulonephritis (MPGN), and mesangial proliferative glomerulonephritis [5]. Typically, MGN is more common in children than adults, whereas MPGN is more common in adults [6]. Various therapeutic approaches for HBV-GN have been tested, including interferon, lamivudine, and entecavir therapy with or without corticosteroid [7–11]. Some case series suggested that antiviral agents may improve proteinuria and renal outcome [12, 13]. However, most of the results are derived from small-scale case series, and a standard of care has not been established yet.

Proteinuria in CHB patients can occur due to HBV-GN but is also a result of primary kidney disease, such as diabetic nephropathy, hypertensive nephropathy, multiple myeloma, or amyloidosis [1]. However, no reports have described the cause of proteinuria among CHB patients. In this study, we aimed to elucidate the causes of proteinuria, as well as the clinical importance and outcome of HBV-GN among Korean CHB patients.

## 2. Patients and Methods

### 2.1. Patients

We retrospectively reviewed the medical records of patients with seropositivity for hepatitis B surface antigen (HBsAg) who underwent renal biopsies for clinically significant proteinuria (>500 mg/day, observed within the last 3 months) from May 2005 to March 2011 at a single tertiary medical center, Seoul National University Hospital (Seoul, Republic of Korea).

Patients with high probability of diabetic nephropathy (i.e., diabetic retinopathy or >10 years' history of diabetes mellitus (DM)) or hypertensive nephropathy (i.e., previous history of hypertensive urgency or crisis) were excluded from this study. Those who had evidence of coinfection with hepatitis C, hepatitis D, and/or human immunodeficiency virus were excluded. The study protocol conformed to the ethical guidelines of the World Medical Association Declaration of Helsinki and was approved by the Institutional Review Board of Seoul National University Hospital (IRB number H-9908-058-003).

### 2.2. The Clinical Data

In every patient we recorded clinical data including age, gender, comorbidities (DM, hypertension), renal histology reports (light microscopy, electron microscopy, and immunohistochemistry), and the use of antiviral agents (interferon or nucleos[t]ide analogues) at the time of kidney biopsy performed.

If a patient was treated with antiviral agents, changes in parameters were determined by collecting data before and after treatment. Routine blood tests included complete blood counts, serum alanine aminotransferase and aspartate aminotransferase, direct and total bilirubin, albumin, cholesterol, blood urea nitrogen (BUN), and creatinine. Laboratory data were obtained every three months from the date of performance of renal biopsy.

Renal function was evaluated using serum creatinine levels and degree of proteinuria. Protein excretion and creatinine clearance were assessed on random spot urine protein-to-creatinine ratio and urine dipstick tests. Urine dipstick measurements were performed using Uropaper alpha III-9L (Shinyang Chemical Co., Ltd., Korea). Urine dipstick results were reported as 0 to 4+ using an automated reader (US-3100R Plus; EIKEN Chemical Co., Ltd., Japan). Urinary protein concentration was graded as follows: − (0 mg/dL), ± (<15 mg/dL), 1+ (15–30 mg/dL), 2+ (30–100 mg/dL), 3+ (100–300 mg/dL), and 4+ (>300 mg/dL). Glomerular filtration rate was assessed using the Cockcroft-Gault formula. The proportion of HBV-GN in CHB patients with proteinuria and change in renal function over time classified by antiviral treatment was evaluated.

### 2.3. Pathological Study

The determination of renal pathology was made by pathologists with at least 10 years' experience. Renal biopsies, all of which contained at least six glomeruli, were processed for light, electron, and immunofluorescent (stained for human immunoglobulins G/M/A, C3, and fibrinogen) microscopy using standard methodologies previously described [14]. Indirect immunofluorescence testing was carried out for HBsAg using the rabbit anti-HBs reagents as the primary antibody [14]. The lesions with glomerular nephritis were classified according to the 1990 WHO Classification Criteria [15]. The diagnosis of HBV-GN was established by serologic evidence of HBV antigens/antibodies, presence of an immune complex glomerulonephritis, immunohistochemical localization of 1 or more HBV antigens, and pertinent clinical history, when available [16].

### 2.4. Statistical Analysis

Statistical analysis was performed using PASW version 18 (IBM; Chicago, IL, USA). Comparisons were made between the intervention group and the control group. Proportions were compared using the Mann-Whitney *U* test, Chi-square test, or Fisher's exact test, where appropriate. A *P value* of less than 0.05 was considered statistically significant.

## 3. Results

### 3.1. Baseline Characteristics

One hundred and twenty-four patients with seropositivity for HBsAg underwent kidney biopsy during the study period. Sixty-nine patients were excluded because kidney biopsy was performed for other reasons aside from proteinuria (e.g., hematuria, acute renal failure, or renal mass). A total of 55 HBsAg positive patients with proteinuria were analyzed in this study. The median age of patients was 51.0 years, and 46 (84%) were males. Six patients (10.9%) had DM, 16 (27.3%) had hypertension, and 13 (23.6%) had concomitant hematuria. The median serum levels of BUN and creatinine were 19.0 mg/dL and 1.2 mg/dL, respectively. The median glomerular filtration rate was 54.2 mL/min, estimated by the Cockcroft-Gault equation. The median value of spot urine protein-to-creatinine ratio was 3.5 mg/g (Table 1).

### 3.2. Distribution of Histological Diagnosis in HBV Patients with Proteinuria

Kidney biopsies were performed on 55 patients with significant proteinuria. The most common final histological diagnoses included MPGN in 13 (23.6%), MGN in 8 (14.5%), and immunoglobulin A nephropathy (IgAN) in 13 (23.6%). Twenty-one patients had other types of GN including focal segmental glomerulosclerosis 3 (5.4%), minimal change disease 3 (5.4%), and diabetic nephropathy 7 (12.7%) (Table 2).

HBV-GN was diagnosed in 20 (36.4%) eligible patients by confirming immune complex deposition: 12 patients (60%) showed MPGN, 7 (35%) showed MGN, and 1 (5%) showed IgAN (Table 2). The remaining 63.6% of patients displayed other types of GN.

### 3.3. Changes in Renal Function among HBV-GN Patients over Time Categorized by Antiviral Treatment

Thirteen of the 20 HBV-GN patients were treated with antiviral agents (the intervention group): 6 with lamivudine, 5 with entecavir, 1 with clevudine, and 1 with pegylated interferon alpha-2a. The median duration of antiviral treatment was 11 months (range, 3–34 months). The remaining 7 patients underwent no antiviral treatment (the control group). Baseline characteristics of both groups are described in Table 1. Baseline HBV DNA level was higher in the intervention group compared with the control group. Otherwise there was no significant difference in baseline characteristics between the two groups. Among the patients who were treated with antiviral agents, 7 patients (54%) showed complete virologic response and 6 (46%) showed partial virologic response. We evaluated the clinical efficacy of treatment by using two outcome measures: changes in serum creatinine and amount of proteinuria (spot urine protein-to-creatinine ratio and dipstick analysis).

**Figure 1:**
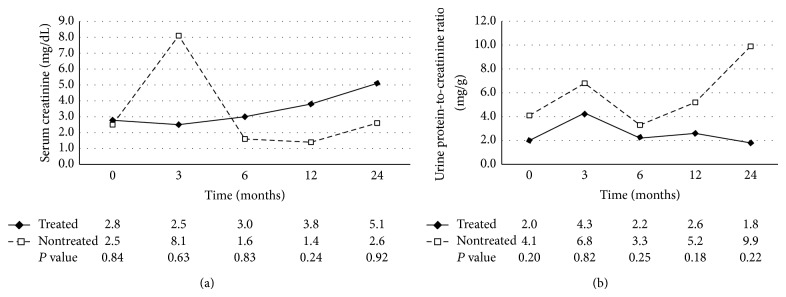
Changes in renal function as determined by serum creatinine level (a) and amount of proteinuria as determined by the urine protein-to-creatinine ratio (b) classified by antiviral treatment over time. Degree of proteinuria was not reduced, and renal function was not significantly improved in the antiviral intervention group when compared to the control group.

In the intervention group, the median value of serum creatinine at baseline and at 3, 6, 12, and 24 months was 2.8, 2.5, 3.0, 3.8, and 3.9 mg/dL, respectively. The value of serum creatinine tended to increase, and there was no significant difference between the intervention and the control groups (Figure 1(a)).

**Figure 2:**
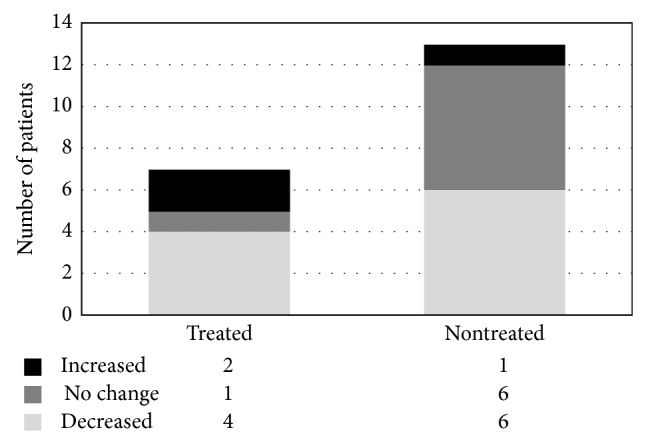
Changes in proteinuria as determined by a urine dipstick test and classified by antiviral treatment. Decrease in proteinuria between the intervention group and the control group was not considered statistically significant (*P* = 0.143).

In the intervention group, the median value of random urine protein-to-creatinine ratio was 2.0 mg/g at baseline, 4.3 mg/g at month 3, 2.2 mg/g at month 6, 2.6 mg/g at month 12, and 1.8 mg/g at month 12. No significant difference was noted in the amount of proteinuria between two groups (Figure 1(b)). The degree of proteinuria was also evaluated by dipstick grading. Each group was divided into three groups based on results of the dipstick protein grading change: increased, decreased, and no significant change. The intervention group showed a 46% decrease in proteinuria whereas the control group showed a decrease of 57%. This differ**e**nce was not considered statistically significant (*P* = 0.143) (Figure 2).

## 4. Discussion

One of the aims of this study was to evaluate the causes of proteinuria among Korean CHB patients and to assess the clinical outcomes of HBV-GN. Our study showed that more than half of the patients with significant proteinuria in CHB were not related to HBV-GN. HBV-GN was diagnosed in 36.4% CHB patients with significant proteinuria and the remaining 63.6% of patients displayed other types of nephropathy aside from HBV-GN. To our knowledge, only a few reports have examined the significance of proteinuria in CHB, and no studies have reported the percentages of HBV-GN. Our study showed that HBV-GN only accounts for approximately one-third of the patients with proteinuria in CHB. The results obtained here suggest that when ascertaining the cause of proteinuria is difficult, kidney biopsy should be considered.

Several reasons may explain the relatively low incidence of HBV-GN in the current study. First, factors other than HBV infection may influence patient outcomes. Huang et al. have reported that DM was the most important factor associated with proteinuria, followed by hypertension, anti-HCV seropositivity, body mass index, age, and triglyceride levels in HBV-infected subjects [17]. Although we excluded some of the confounding comorbidities, patients with DM and hypertension accounted for 10.9% and 27.3% of the patients enrolled in the study, respectively. Consequently, these diseases may be responsible for the proportion of HBV-GN observed. These findings suggest that HBV-GN may be considered as the cause of proteinuria in CHB, but metabolic abnormalities, such as DM, hypertension, obesity, and dyslipidemia, as well as age, should also be considered.

Histological diagnosis is mandatory for the diagnosis of HBV-GN, but it is not easily distinguished from forms. Comparison of idiopathic GN with HBV-GN is difficult, but some differences were noted. First, mesangial deposits were more frequently noted in patients with HBV-associated MGN when compared to others with idiopathic MGN [14]. Second, most of the children with HBV-associated MGN are reported to be characterized by the histological features of MPGN in addition to those of idiopathic MGN [18]. In general, HBV-GN displayed a wide morphological spectrum along with overlapping ultrastructural features in MGN and MPGN when compared to typical MGN and MPGN [19]. Another distinguishing characteristic of HBV-GN is that the subepithelial immune deposits are frequently associated with subendothelial or mesangial deposits [18, 20]. This is in contrast to idiopathic MGN, where deposits are seen in a predominantly subepithelial location [21]. Pathologists distinguish HBV-GN from idiopathic GN with these findings, but interobserver discrepancy still exists between pathologists. Although the renal pathology of our study is based on the judgment of expert pathologists, diagnosis was made by different pathologists, so different conclusions might have been reached had other pathologists been consulted.

The results of histological diagnosis of HBV-GN showed that MPGN was most common, followed by MGN and IgAN. These results correspond to those of earlier studies. Kim et al. reported that the frequency of MPGN was 42.6%, MGN 35.5%, mesangial glomerulonephritis 20.0%, and minimal change disease 18.8% [22]. Lee et al. reported that MPGN was most common, followed by MGN and IgAN [14]. Yoon et al. reported that the frequency of MGN was 35.7%, mesangial glomerulonephritis 28.6%, MPGN 25%, and minimal change disease 10.7% [23].

Another major finding of this study was that antiviral therapy against HBV infection failed to improve the renal prognosis of HBV-GN. Consequently, we reached the conclusion that the degree of proteinuria was not reduced, and renal function was not significantly improved in the antiviral intervention group when compared to the control group, although more than half of the intervention group showed complete virologic response. These findings contrast with the results of previous studies, which indicated that most patients with HBV-GN were successfully treated by antiviral therapy and that the overall estimate for remission of nephrotic syndrome was more than 60% [13, 24]. We suppose that the following factors may contribute to the outcome. First, a retrospective study design and small sample size may have caused this negative result. Besides, heterogeneous antiviral regimens and doses may have also confounded the effect of treatment. Second, the fact that most patients were adults may be another possible reason for failure of antiviral treatment to improve renal function. The natural history of HBV-associated nephrotic syndrome shows gradual improvement in younger patients, whereas, in adults, it generally continues to progress with a low remission rate [25, 26]. The median age of patients in the present study was 48.3 years and they are thought to be acquiring chronic HBV infection in perinatal period. In these patients, HBV DNA integrates into host-cell chromosomal DNA and this makes the antiviral therapy less effective. If we had studied more patients including children, the results may have been different. Thus, well-designed prospective studies in younger patients are warranted. Third, there is a possibility that antiviral therapy might not be effective when irreversible structural changes in the glomeruli and immune deposit exist. In our study, all the patients showed complete or partial virologic response, but the clinical efficacy of antiviral agents of renal function was disappointing. It is assumed that immune deposit and irreversible change still persist even in the absence of circulating serum HBV DNA, and this reduces the effect of treatment.

In summary, our study showed that HBV-GN was diagnosed in 36.4% of CHB patients with significant proteinuria, while the remaining 63.6% had other types aside from HBV-GN. The most common histological diagnosis of HBV-GN was MPGN, followed by MGN and IgAN. Antiviral treatment failed to demonstrate either improvement of renal function or reduction in proteinuria.

In conclusion, proteinuria among CHB patients in Korea can be caused by various entities. HBV-GN is not a single predominant cause of proteinuria in CHB, and metabolic abnormalitiesshould also be considered. Insufficient evidence is available to confirm whether antiviral therapy against HBV infection may improve the renal prognosis of HBV-GN, and further well-designed prospective studies are needed.

## Figures and Tables

**Table 1:** Baseline characteristics of total patients (*n* = 55) and HBV-GN patients (*n* = 20) categorized by antiviral treatment subgroups.

Characteristics	Total (*n* = 55)	Intervention group (*n* = 13)	Control group (*n* = 7)	*P* value
Age (years)^+^	51.0 (24.0–75.0)	49.0 (32.0–75.0)	53.0 (24.0–70.0)	0.28^a^
Male sex^++^	46 (84)	9 (69.2)	7 (100)	0.44^b^
Diabetes mellitus^++^	6 (10.9)	3 (23.0)	2 (28.6)	0.88^b^
Hypertension^++^	15 (27.3)	4 (30.8)	3 (42.9)	0.70^b^
Hematuria^++^	13 (23.6)	2 (15.4)	0 (0)	0.59^b^
Liver cirrhosis^∗++^	22 (40)	5 (38.5)	3 (42.9)	0.61^b^
Child-Pugh class^++^				0.54^b^
Class A	12 (21.8)	3 (23.1)	2 (28.6)	
Class B	10 (18.2)	2 (15.4)	1 (14.3)	
Laboratory assessments				
Baseline HBsAg positive^++^	55 (100)	13 (100)	7 (100)	
Baseline anti-HBs positive^++^	0 (0)	0 (0)	0 (0)	
Baseline HBeAg positive^++^	21 (38.2)	7 (53.8)	4 (57.1)	0.53^b^
Baseline anti-HBe positive^++^	34 (61.8)	6 (46.1)	3 (42.9)	0.53^b^
Baseline HBV DNA level^++^				<0.001^b^
0–2000 IU/mL	22 (40.0)	3 (23.1)	4 (57.1)	
2000–20000 IU/mL	4 (7.3)	2 (15.4)	1 (14.3)	
>20000 IU/mL	29 (52.7)	8 (61.5)	2 (28.6)	
Serum BUN (mg/dL)^+^	19.0 (9.0–92.0)	24.0 (9.0–92.0)	18.0 (13.0–66.0)	0.76^a^
Serum Cr (mg/g)^+^	1.2 (0.7–6.6)	1.3 (0.7–6.6)	1.1 (0.8–2.7)	0.88^a^
Urine protein-to-creatinine ratio (mg/g)^+^	3.5 (0.8–10.6)	3.4 (0.8–10.6)	3.8 (3.0–5.6)	0.38^a^
Serum cholesterol (mg/dL)^+^	204.0 (91.0–447.0)	202.0 (91.0–447.0)	240.0 (105.0–273.0)	0.88^a^
Total protein (g/dL)^+^	5.9 (4.3–7.5)	5.9 (4.3–7.5)	6.0 (4.4–6.8)	0.59^a^
Serum albumin (g/dL)^+^	2.9 (1.9–4.5)	2.8 (1.9–4.5)	3.7 (2.0–4.4)	0.70^a^
Total bilirubin (mg/dL)^+^	0.7 (0.3–4.7)	0.8 (0.3–4.7)	0.7 (0.5–1.5)	0.40^a^
Alkaline phosphatase (IU/L)^+^	66.0 (27.0–308.0)	67.0 (33.0–308.0)	66.0 (27.0–116.0)	0.49^a^
AST (IU/L)^+^	29.0 (10.0–198.0)	30.0 (10.0–198.0)	28.0 (19.0–33.0)	0.54^a^
ALT (IU/L)^+^	35.0 (7.0–179.0)	38 (7.0–179.0)	33.0 (13.0–72.0)	0.44^a^

^*^Liver cirrhosis was diagnosed when the platelet count was below 100,000/mm^3^ and associated splenomegaly or esophageal-gastric varices were detected.

^+^Data are medians, and data in parentheses are ranges.

^++^Data are numbers of patients and data in parentheses are percentages.

^a^Mann-Whitney *U* test was used to analyze the differences between the intervention group and control group.

^b^ *χ* ^2^ test (or Fisher's exact test) was used to analyze the differences between the intervention group and control group.

**Table 2:** Frequency of HBV-GN among patients with proteinuria and hepatitis B and diagnostic distribution of renal pathologies in HBV-GN patients.

Histological diagnosis	All numbers	HBV-GN
Membranoproliferative glomerulonephritis	13	12
Membranous glomerulonephritis	8	7
IgA nephropathy	13	1
Focal segmental glomerulosclerosis	3	0
Minimal change disease	3	0
Diabetic nephropathy	7	0
Others	8	0
Total	**55**	**20**
